# Directly targeting c-Myc contributes to the anti-multiple myeloma effect of anlotinib

**DOI:** 10.1038/s41419-021-03685-w

**Published:** 2021-04-14

**Authors:** Yang Cao, Huizhuang Shan, Meng Liu, Jia Liu, Zilu Zhang, Xiaoguang Xu, Yue Liu, Hanzhang Xu, Hu Lei, Miao Yu, Xingming Zhang, Wanting Liu, Zhilei Bu, Zhixiao Fang, Yanjie Ji, Hua Yan, Weiying Gu, Yingli Wu

**Affiliations:** 1 Department of Hematology, The First People’s Hospital of Changzhou, Third Affiliated Hospital of Soochow University, 213003 Changzhou, Jiangsu Province P.R. China; 2 Hongqiao International Institute of Medicine, Shanghai Tongren Hospital/Faculty of Basic Medicine, Chemical Biology Division of Shanghai Universities E-Institutes, Key Laboratory of Cell Differentiation and Apoptosis of the Chinese Ministry of Education, Shanghai Jiao Tong University School of Medicine, 200025 Shanghai, China; 3 Department of Hematology, Ruijin Hospital, Shanghai Jiao Tong University School of Medicine, 200025 Shanghai, China

**Keywords:** Myeloma, Target identification

## Abstract

Despite the significant advances in the treatment of multiple myeloma (MM), this disease is still considered incurable because of relapse and chemotherapy resistance, underscoring the need to seek novel therapies with different mechanisms. Anlotinib, a novel multi-targeted tyrosine kinase inhibitor (TKI), has exhibited encouraging antitumor activity in several preclinical and clinical trials, but its effect on MM has not been studied yet. In this study, we found that anlotinib exhibits encouraging cytotoxicity in MM cells, overcomes the protective effect of the bone marrow microenvironment and suppresses tumor growth in the MM mouse xenograft model. We further examined the underlying molecular mechanism and found that anlotinib provokes cell cycle arrest, induces apoptosis and inhibits multiple signaling pathways. Importantly, we identify c-Myc as a novel direct target of anlotinib. The enhanced ubiquitin proteasomal degradation of c-Myc contributes to the cell apoptosis induced by anlotinib. In addition, anlotinib also displays strong cytotoxicity against bortezomib-resistant MM cells. Our study demonstrates the extraordinary anti-MM effect of anlotinib both in vitro and in vivo, which provides solid evidence and a promising rationale for future clinical application of anlotinib in the treatment of human MM.

## Introduction

Multiple myeloma (MM) is a hematologic malignancy characterized by the accumulation of clonal plasma cells predominantly in bone marrow^1^. Of all the hematologic malignancies, MM accounts for about 1.8%, ranking third in the hematological malignant tumors after lymphoma and leukemia^2^. Over the last few decades, the advents of novel treatments, such as immunomodulatory drugs (e.g., lenalidomide, pomalidomide)^3^, proteasome inhibitors (e.g., bortezomib, carfilzomib)^4^, histone deacetylase inhibitors (e.g., panobinostat)^5^, monoclonal antibodies (e.g., daratumumab, elotuzumab)^6^, chimeric antigen receptor T-cell (CAR-T) therapy^7^, and autologous transplantation^8^, have transformed the management for MM patients and improved the therapeutic outcomes. However, relapse or chemotherapy resistance is an expected part of the disease course, and MM heretofore has still been considered fatal and incurable^9^. Therefore, studies aiming to uncover new or alternative therapies are urgently needed to improve the clinical outcomes of MM.

Epidemiologic studies have demonstrated that symptomatic MM is consistently preceded by a precursor state named monoclonal gammopathy of undetermined significance (MGUS), followed by smoldering multiple myeloma (SMM)^10^. Of note, unlike what is often present in MM patients, the translocations involving the *MYC* locus and the gains of *MYC* are rarely detected in patients with MGUS and SMM^11,12^. In both the transition of MGUS to MM and the late stages of MM progression, c-Myc is frequently activated and proposed to be a significant trigger^13,14^. c-Myc exerts neoplastic effects by integrating various signals to affect a diverse array of cellular processes, including DNA replication, cell growth, metabolism, cell cycle progression, and so on^15–17^. Since c-Myc dysregulation is a unique feature in the genetic landscape of MM, it represents an attractive drug target for MM treatment^18^. Until now, several strategies have been developed to target MYC in MM. However, many of these approaches are suffered from low potency and poor pharmacokinetic properties. Besides, direct inhibition of c-Myc in the area of ligand discovery remains a challenge.

Anlotinib (AL3818) is a novel oral multi-targeted receptor tyrosine kinase inhibitor (TKI) that targets vascular endothelial growth factor receptor (VEGFR) 1-3, c-Kit, platelet-derived growth factor receptor (PDGFR)-α/β, and fibroblast growth factor receptor (FGFR) 1–4. Anlotinib is considered as a broad-spectrum drug with inhibitory effects on tumor progression, apoptosis, proliferation, and angiogenesis^19^. Based on the result of phase 3 randomized clinical trial (ALTER-0303), anlotinib has become the first drug approved in China for a third-line treatment or beyond for advanced non-small cell lung carcinoma (NSCLC)^20^. Recent preclinical and ongoing clinical trials have demonstrated promising antitumor activities of anlotinib against diverse malignant tumor types (e.g., metastatic renal cell carcinoma^21^, advanced medullary thyroid carcinoma^22^, and soft tissue sarcoma^23^); however, no research has thrown light on its potential efficacy in MM.

This study is the first to report the therapeutic effects of anlotinib on MM and explore its possible molecular mechanisms. We found that anlotinib exerts potent anti-MM activity in vitro and in vivo, regardless of the bone marrow microenvironment protection or bortezomib-resistance. Further studies demonstrate that anlotinib directly interacts with c-Myc, accelerates its ubiquitin proteasome-dependent degradation, followed by genome-wide downregulation of c-Myc-dependent target genes, which contributes to the anti-MM effect of anlotinib. Our work provides the proof-of-concept for clinical evaluation of anlotinib in the treatment of MM.

## Materials and methods

### Cell culture and reagents

The human MM cell lines NCI-H929, RPMI-8226, MM.1S, and U266 were purchased from the American Type Culture Collection (Manassas, VA, USA). LP1 and OPM2 were obtained from the German Collection of Microorganisms and Cell Cultures (Braunschweig, Germany). The cell lines were authenticated by short-tandem repeat profiling prior to use and were routinely tested negative for mycoplasma contamination. The bortezomib-resistant cells, NCI-H929-BR, and MM.1S-BR, were induced by ongoing treatment of bortezomib. Bone marrow mononuclear cells (BMMCs) were isolated from three MM patients and four healthy donors using Ficoll-Hypaque density gradient centrifugation. CD138^+^ MM cells were purified from the BMMCs of MM patients by Human CD138 MicroBeads (Miltenyi Biotech, Auburn, CA). Bone marrow mesenchymal stem cells (BMSCs) were generated from BMMCs of MM patients. Informed consent was obtained in accordance with the Declaration of Helsinki protocol and approval by the Institutional Review Board of The Third Affiliated Hospital of Soochow University. All the cells were cultured in a humidified chamber at 37 °C in 5% CO_2_ atmosphere, suspended in RPMI 1640 (MM cell lines) or DMEM medium (BMSCs), and added 10% fetal bovine serum (Sigma-Aldrich, St.Louis, MO), 100 μg/ml streptomycin and 100 IU/ml penicillin (Invitrogen).

Anlotinib, CHX and MG-132 were purchased from CSNpharm (Chicago, IL, USA), dissolved in Dimethyl Sulfoxide (Sigma, St. Louis, MO) and stored in dark at −20 °C until use. Recombinant human interleukin 6 (IL-6) was obtained from R&D Systems (Minneapolis, MN). Matrigel purchased from Corning Life Sciences (Corning, NY, USA).

### Cell viability assay

The Cell Counting Kit-8 (CCK-8, Dojindo, Kumamoto, Japan) assay was used to measure the cell viability according to the manufacturer’s instructions. Briefly, the cells were cultured in 96-well plates, treated with drugs at the indicated concentrations and then incubated with CCK-8 working solution for 1–4 h at 37 °C. The resulting absorbance was detected at 450 nm in a microplate reader. The combined effect was assessed using the CompuSyn software (Biosoft, Ferguson, MO, USA). The combination index (CI) < 1, =1, and >1 indicated synergism, addictive effect, and antagonism, respectively.

### Flow cytometric analysis for cell cycle and apoptosis

For cell cycle distribution, cells were harvested, fixed with 70% ethanol overnight at −20 °C and incubated with PI/RNase staining buffer (BD Biosciences) in the dark. The fluorescence of the stained cells was measured using a FACScan flow cytometer (Becton Dickinson, San Diego, CA, USA). Cell apoptosis was determined by flow cytometry after staining with Annexin V and PI detection kit (BD Biosciences, San Diego, CA, USA). Apoptotic cells include early (Annexin V positive and PI negative) and late (both Annexin V and PI positive) apoptosis.

### TUNEL assay and DAPI staining

RPMI-8226 and NCI-H929 cells were treated with 5 μM anlotinib for 24 h. After fixed in 4% paraformaldehyde and permeabilized with 0.3% Triton X-100, cells were incubated with TUNEL detection solution (50 μl/well; Beyotime Institute of Biotechnology) for 60 min and then DAPI for 5 min at 37 °C. The cells were imaged under confocal microscopy (Nikon, Japan).

### Western blotting

Cells were harvested, washed, and lysed. Equal quantities of protein extract were electrophoresed using sodium dodecyl sulfate–polyacrylamide gel electrophoresis and transferred to nitrocellulose membranes. After blocking with 5% non-fat milk, the membranes were incubated with primary antibodies overnight at 4 °C, followed by incubation with the horseradish peroxidase (HRP)-conjugated secondary IgG antibody. The signals were visualized using the ECL detection system (Thermo Fisher, USA). Antibodies against PARP1 (#9532), caspase 3 (#9662) and 9 (#9502), p-ERK1/2 (T202/Y204, #4370), ERK1/2 (#4695), p-AKT (S473, #4060), AKT (#4691), p-mTOR (S2448, #5536), mTOR (#2983), p-STAT3 (Y705, #9145), STAT3 (#4904), p-p65 (S536, #3033), p65 (#8242), MAX (#4239), and β-actin (#4970) were purchased from Cell Signaling Technology (Beverly, MA, USA); c-Myc (#ab32072), p-c-Myc (T58, #ab28842), and p-c-Myc (S62, #ab51156) was from Abcam (Cambridge, UK).

### Quantitative reverse transcription real-time PCR (qRT-PCR)

Total RNA of the agent-treated cells was extracted using TRIzol reagent (Invitrogen). Thereafter, the cDNA was synthesized using a cDNA reverse transcription kit (TransGen Biotech, Beijing, China). The qRT-PCR was performed in triplicate on 7900HT real-time PCR instrument (Applied Biosystems, CA, USA) using the SYBR Green Mix (Applied Biosystems). The primer sequences of c-Myc were as follows: forward primer, CTGCGTAGTTGTGCTGATGT; reverse primer, ATCATTTCCATGACGGCCTGT. The relative expression level of c-Myc was normalized to β-actin and then presented by the 2^−ΔΔCt^.

### Mouse xenograft model

The BALB/c nude mice (5-weeks old) were purchased from the Shanghai Laboratory Animal Center (Shanghai, China). The animal experiments were approved by the Animal Research Committee of The Third Affiliated Hospital of Soochow University and were performed in accordance with established guidelines. Mice were bred in the specific pathogen-free (SPF) grade of the animal care facility and a 12 h dark–light cycle. After acclimatization, 1 × 10^7^ NCI-H929 cells were mixed with Matrigel and subcutaneously injected into the right flank of the mice. When tumors were measurable, mice were randomly divided into treatment and control groups (*n* = 6 mice each). In the treatment group, mice received 3 mg/kg anlotinib orally daily for consecutive 14 days. Tumor size and mouse body weight were monitored every other day. Tumor volume (mm^3^) was calculated as length × width^2^/2. After 14 days of treatment, all the mice were killed, and the tumor tissues were weighed and prepared for western blotting and immunohistochemistry (IHC).

### Lentiviral packaging

Lentiviral vectors were co-transfected with packaging plasmids psPAX2 and pMD2G into HEK293T cells. Infectious lentiviruses were harvested and filtered through 0.45 μm PVDF filters. Recombinant lentiviruses were concentrated 100-fold by ultracentrifugation (2 h at 120,000×*g*). MM cells were prepared and infected by lentiviruses, and western blotting was used to validate the expression of c-Myc. The shRNA sequences of c-Myc were: sh-1#: 5′-CCTGAGACAGATCAGCAACAA-3′ and sh-2#: 5′-CCTGAGACAGATCAGCAACAA-3′.

### RNA sequencing (RNA-seq)

Total RNA was extracted from the NCI-H929 cells after treated DMSO or anlotinib for 12 h using Trizol Reagent (Invitrogen, USA). Then, the RNA samples were sent to Gminix, Biotechnology Co, Ltd (Shanghai, China) for RNA-seq on an Illumina HiSeq ×10 sequencing platform with 10G reads. The procedures were performed as described in detail on the website of Gminix. The raw data for this study is available at the NCBI sequence read archive (SRA) database under the accession number: PRJNA587019.

### Preparation of recombinant wild-type c-Myc protein

Human full-length c-Myc were cloned into a pET-30c vector containing a 6 × His tag at the C-terminal region. The *E. coli* strain BL21 was transformed with pET-c-Myc, cultured in 2 × YT and induced with 0.2 mM IPTG for 6 h. After centrifugation, the supernatant was applied to a Ni-beads column and washed with wash buffer. Proteins were eluted with elution buffer.

### Cellular thermal shift assay (CETSA)

CETSA is a newly developed approach based on the thermal stabilization of ligand-bound proteins. The procedure of CETSA has been described previously^24^. Briefly, cell lysates from NCI-H929 cells or purified c-Myc proteins were incubated with anlotinib or DMSO for 30 min, and then heated at different temperatures for 3 min. The mixtures were centrifuged, and the supernatants were subjected to western blotting.

### Drug affinity responsive targets stability (DARTS)

DARTS was conducted in NCI-H929 cells following the previously published protocol^25^. Briefly, the cell lysates were prepared with lysis buffer and the concentration was determined as described above. The cell lysates were incubated with anlotinib or DMSO for 1 h at room temperature, and then digested with Pronase (Roche) for an additional 20 min at room temperature. The reaction was quenched by the protein inhibitor cocktail and the samples were subjected to western blotting.

### Co-immunoprecipitation (Co-IP)

Co-IP was conducted to detect the protein interaction. Briefly, whole cell lysates were incubated with indicated antibodies and Protein G/A beads overnight at 4 °C. After four times wash with RIPA buffer, beads were boiled in loading buffer to elute protein complexes, followed by western blotting with specific antibodies. HRP-conjugated AffiniPure Mouse Anti-Rabbit IgG Light Chain was purchased from ABclonal (China, #AS061).

### Statistical analysis

Statistical analysis was performed using SPSS version 22.0 software (IBM Corporation, Chicago). The quantitative data are shown as the mean ± SD. The data were examined using the two-tailed Student’s *t*-test or one-way ANOVA with post-hoc Bonferroni correction. The IC_50_ value was calculated based on the dose-response curve using GraphPad Prism 8.0 software. Statistical significance is indicated by *P < 0.05, **P < 0.01, and ***P < 0.001.

## Results

### Anlotinib exhibits potent cytotoxicity against MM cell lines and primary cells

**Fig. 1:**
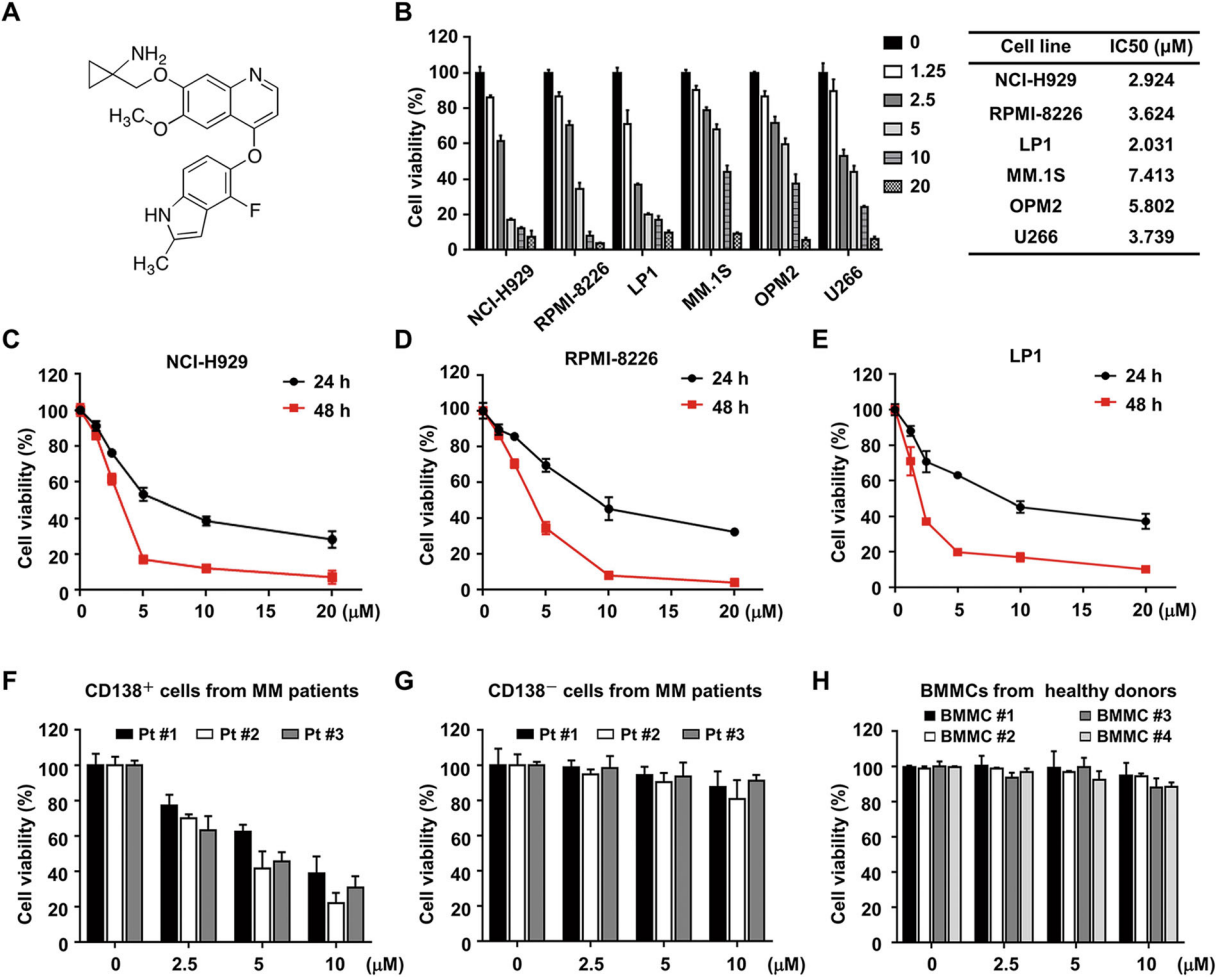
Anlotinib exerts potent cytotoxicity against MM cells. **A** Chemical structure of anlotinib. **B** MM cell lines (NCI-H929, RPMI-8226, LP1, MM.1S, OPM2, and U266) were treated with anlotinib (0–20 μM) for 48 h, and the cell viability was detected by CCK8. The IC50 value was calculated based on the dose–response curve using Prism 8.0 software. **C**–**E** NCI-H929, RPMI-8226 and LP1 cells were treated with anlotinib for 24 and 48 h, followed by the assessment of cell viability. **F** The CD138^+^ and **G** CD138^−^ cells isolated from MM patients were treated with anlotinib (0–10 μM) for 24 h, and then the cell viability was assessed. **H** Normal BMMCs from four healthy donors were treated with anlotinib (0–10 μM) for 24 h. Data are shown as mean ± SD and representative of three independent experiments.

To determine whether anlotinib (Fig. 1A) has a cytotoxic effect on human MM cells, we conducted CCK-8 assay in a panel of MM cell lines (NCI-H929, RPMI-8226, LP1, MM.1S, OPM2, and U266). As shown in Figs. 1B and S1, anlotinib-induced dose-dependent cytotoxicity in all MM cell lines with IC_50_ values ranging from 2.0 to 7.4 μM. We also treated NCI-H929, RPMI-8226, and LP1 cells with anlotinib (0–20 μM) for 24 and 48 h, and observed that the cell viabilities were inhibited in a time-dependent manner (Fig. 1C–E). Moreover, the anti-MM effect of anlotinib was also observed in CD138^+^ MM cells isolated from BMMCs of three MM patients (Fig. 1F). In contrast, anlotinib had a low or no cytotoxic effect on CD138^−^ cells from MM patients or BMMCs from healthy donors (Fig. 1G, H). These findings indicated that anlotinib has potent cytotoxicity in MM cell lines and primary cells, but has a faint effect on normal cells.

### Anlotinib antagonizes the protective effect of bone marrow microenvironment on MM cells

**Fig. 2:**
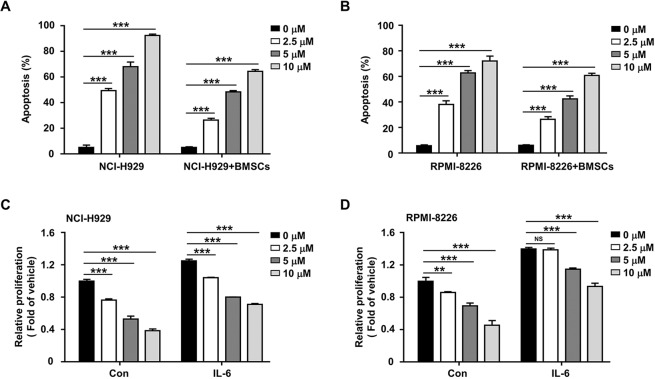
Anlotinib overcomes the protective effect of the BMSCs and exogenous IL-6 on MM cells. **A**, **B** NCI-H929 and RPMI-8226 cells were co-cultured with or without BMSCs, and then treated with anlotinib (0–10 μM) for 48 h. After staining with Annexin V and PI, flow cytometry analysis was performed to assess the apoptosis rate. **C**, **D** NCI-H929 and RPMI-8226 cells were treated with anlotinib (0–10 μM) for 48 h, in the presence or absence of IL-6 (10 ng/ml). Cell proliferation was measured by CCK-8 assay. **P < 0.01; ***P < 0.001; ^NS^*P* > 0.05. Data are shown as mean ±SD and from three independent experiments.

Given that the bone marrow microenvironment promotes the proliferation, survival, and chemoresistance of MM cells, we explored whether anlotinib could overcome the protective effect of bone marrow microenvironment. First, we detected the cytotoxicity of anlotinib on NCI-H929 and RPMI-8226 cells when co-cultured with patient-derived BMSCs. Notably, anlotinib induced remarkable apoptosis of MM cells even in the presence of BMSCs (Fig. 2A, B), while had no significant cytotoxicity in BMSCs (Fig. S2). In addition, the bone marrow microenvironment was mimicked by culturing MM cells in the presence of IL-6. As shown in Fig. 2C, D, although IL-6 effectively promoted the proliferation of MM cells, anlotinib still induced cytotoxicity in both NCI-H929 and RPMI-8226 cells. These data suggested that anlotinib not only directly targets MM cells, but also partially overcomes the proliferative and anti-apoptotic effects mediated by the MM-host bone marrow microenvironment.

### Anlotinib induces cell cycle arrest and apoptosis in MM cells

**Fig. 3:**
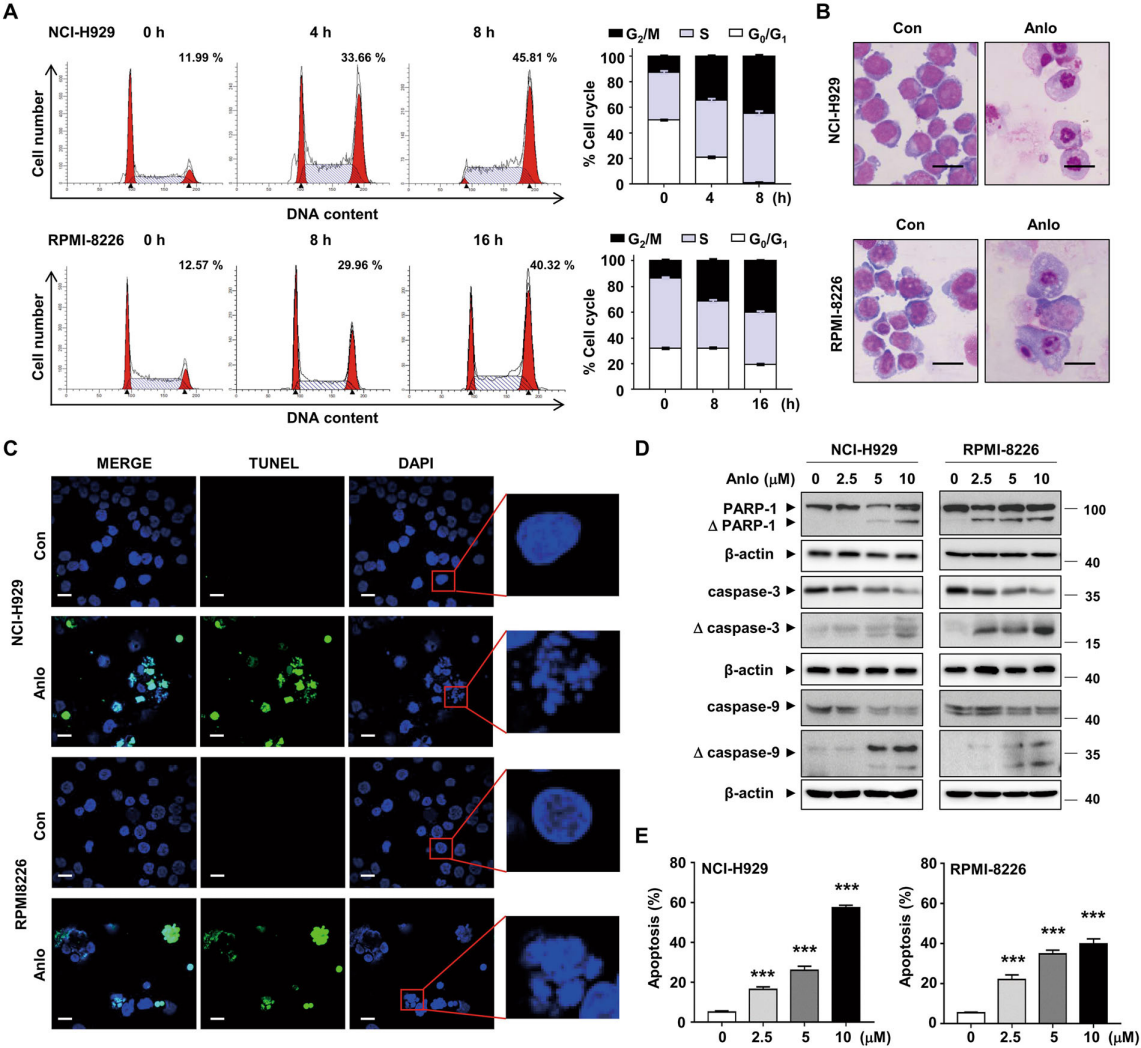
Anlotinib induces cell cycle arrest and apoptosis in MM cells. **A** NCI-H929 and RPMI-8226 cells were treated with 5 µM anlotinib for the indicated time. Cell cycle was analyzed by flow cytometry. The numerical percentage shown indicates the G_2_/M population. **B** The morphology of NCI-H929 and RPMI-8226 cells after anlotinib treatment (5 μM, 24 h) was observed by Wright staining. Scale bar: 20 μm. **C** Representative images of TUNEL and DAPI staining for cells treated with anlotinib (5 μM, 24 h). The green dot represents positive TUNEL staining. The blowup images from the red boxes showed a significant fraction of nuclei after anlotinib exposure. Scale bar: 20 μm. **D** NCI-H929 and RPMI-8226 cells were treated with anlotinib (0–10 μM) for 24 h. Whole cell lysates were subjected to western blotting using antibodies against PARP-1, caspase 3, caspase 9, and β-actin. **E** Cells were treated as described in **D**, and apoptosis was detected by flow cytometry using Annexin V/PI staining. The percentages of apoptotic cells were shown in the histogram from three independent experiments. Each experiment was performed in triplicate. Con: control group; Anlo: anlotinib group. ***P < 0.001.

Since cell growth is closely related to cell cycle progression, we evaluated the cell cycle distribution of NCI-H929 and RPMI-8226 cells treated with anlotinib. The results showed that anlotinib caused a significant accumulation of cells in the G_2_/M phase (Fig. 3A). The morphological staining revealed increased cell fragments and debris in anlotinib-treated MM cells, which indicated the potential impact of anlotinib on cell apoptosis (Fig. 3B). Nuclear morphology was assessed by DAPI staining, and showed the formation of the dot-like apoptotic body in anlotinib-treated cells, while the nucleus of control cells was round in shape (Fig. 3C). In addition, TUNEL-positive cells increased strikingly in NCI-H929 and RPMI-8226 cells after anlotinib treatment (Fig. 3C). Consistent with these findings, anlotinib induced cleavage of caspase 3 and 9, as well as PARP-1 (Fig. 3D). As assessed by flow cytometry, anlotinib treatment resulted in a dose-dependent increase in Annexin V-positive MM cells (Fig. 3E). Thus, these data demonstrated that anlotinib induces cell cycle arrest and apoptosis of MM cells.

### Genome-wide transcriptome analysis indicates c-Myc as a key regulator of anlotinib-treated MM cells

**Fig. 4:**
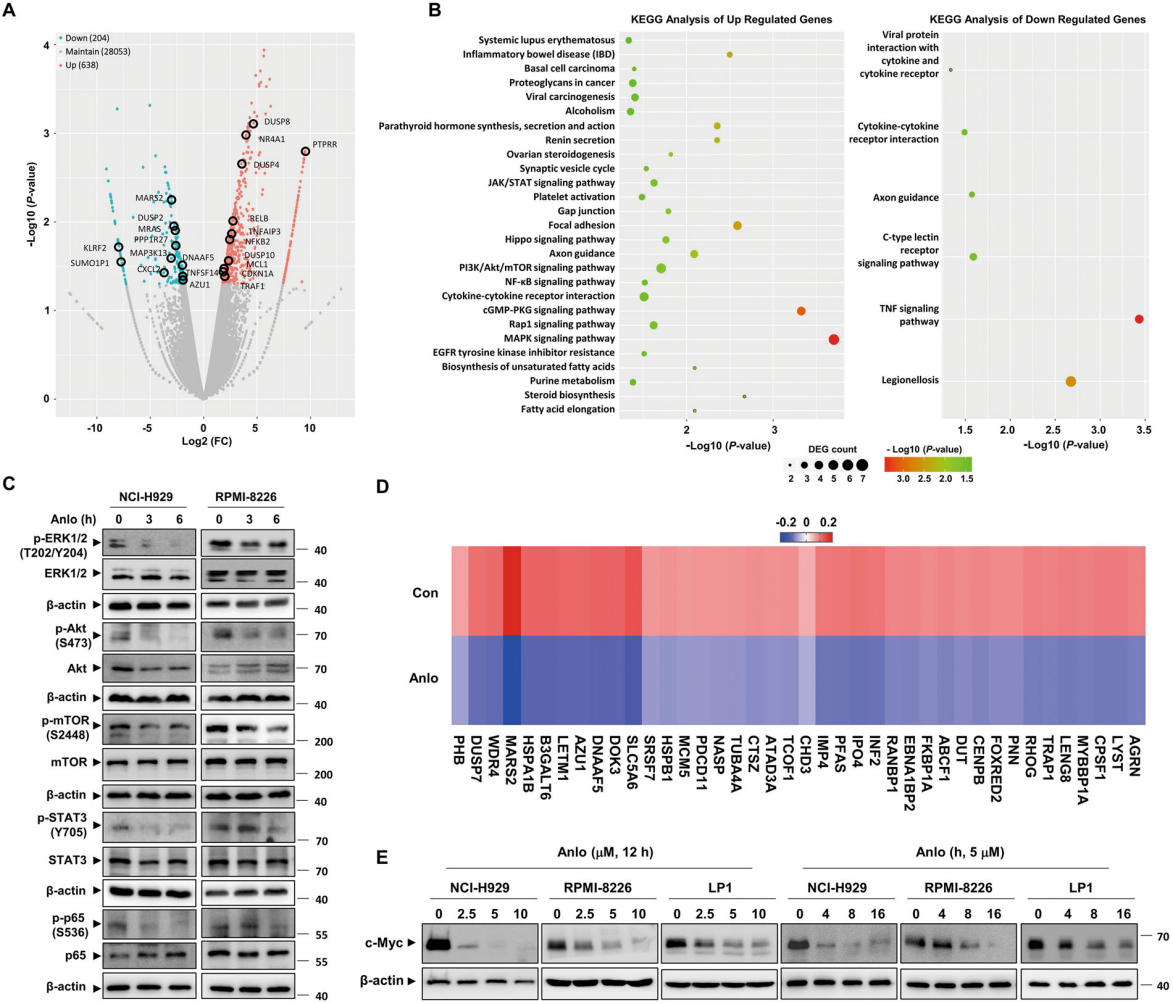
Genome-wide transcriptome analysis of anlotinib-treated MM cells. **A** Volcano plot of the DEGs from the transcriptomes of the control and anlotinib group (*P* < 0.05, Fold change > 1.5). **B** KEGG analysis of the DEGs. **C** NCI-H929 and RPMI-8226 cells were treated with anlotinib (5 μM) for the indicated time. Whole cell lysates were subjected to western blotting. **D** Heatmaps of the top 40 downregulated c-Myc target genes in NCI-H929 cells treated with anlotinib versus DMSO for 12 h. Rows show *Z*-scores are calculated for each cell type. **E** NCI-H929, RPMI-8226, and LP1 cells were treated with the indicated concentration of anlotinib for 24 h or 5 μM anlotinib for the indicated time. Whole cell lysates were subjected to western blotting using c-Myc and β-actin antibodies. Con control group, Anlo anlotinib group. The experiments were performed in triplicate.

Based on the observations of anti-MM effects exerted by anlotinib, we employed RNA-Seq technology to explore the underlying molecular mechanisms. A total of 842 differentially expressed genes (DEGs) were identified (Fig. 4A) and subjected to Kyoto Encyclopedia of Genes and Genomes (KEGG) analysis. The results revealed that the DEGs were mainly involved in the multiple signaling pathways which are important for MM cell proliferation and survival (Fig. 4B). Consistent with the results of RNA-Seq, we observed inhibited MAPK, PI3K/Akt/mTOR, JAK/STAT, and NF-κB signaling in anlotinib-treated NCI-H929 and RPMI-8226 cells (Fig. 4C). It is widely recognized that oncogene c-Myc has a vital role in the pathophysiology of MM and is regulated by a large network of interconnected signaling pathways involves MAPK, PI3K/Akt, JAK/STAT, and NF-κB^26–29^. Intriguingly, further analysis of the DEGs heatmap showed that the genes associated with the c-Myc targets were markedly downregulated by anlotinib treatment (Fig. 4D). Then, we examined the levels of c-Myc protein in three MM cell lines after anlotinib treatment, and the results showed that anlotinib dramatically down-regulated c-Myc in a dose- and time-dependent manner without effect on its mRNA levels (Figs. 4E and S3). Thus, these results indicated that anlotinib reduces c-Myc protein probably at the post-transcriptional level.

### Anlotinib directly targets c-Myc and promotes its ubiquitin proteasomal degradation

**Fig. 5:**
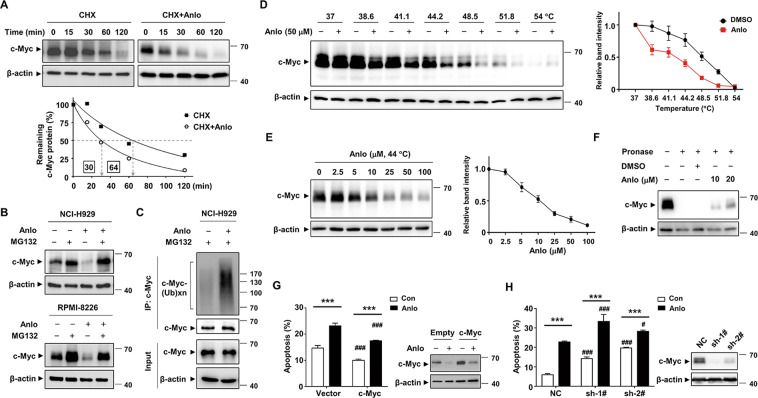
Anlotinib directly targets c-Myc and promotes its ubiquitin proteasomal degradation. **A** NCI-H929 cells were treated with CHX (10 μg/ml) in the presence or absence of anlotinib (5 μM) for the indicated time. The levels of c-Myc protein were evaluated and plotted in the graph. **B** NCI-H929 and RPMI-8226 cells were treated with either anlotinib (5 μM) and/or MG132 (5 μM) for 6 h, and then the c-Myc protein was assessed by western blotting. **C** NCI-H929 cells were pretreated with 5 μM MG132 for 3 h and then incubated with 5 μM anlotinib for 3 h. The cell lysates were immunoprecipitated with anti-c-Myc antibody and then probed with anti-ubiquitin antibody. **D**, **E** The binding between anlotinib and c-Myc protein was examined by the CETSA method at different temperatures or doses. The indicated proteins were evaluated by western blotting (left). CETSA curves of c-Myc were determined in the absence and presence of anlotinib. Each band intensity of c-Myc in DMSO or anlotinib group was normalized with respect to that obtained at the lowest temperature or dose (right). **F** Whole cell lysates of NCI-H929 cells were incubated with anlotinib followed by digestion with pronase according to the “Materials and methods” section. Then, the degree of c-Myc degradation was determined by western blot. **G**, **H** Overexpression and shRNA knockdown efficiency of c-Myc in NCI-H929 cells was measured by western blotting analysis. The effects of c-Myc knockdown or overexpression on cell apoptosis were evaluated by flow cytometry. The presented columns are given as the means ± SD. Statistically significant values compared with the DMSO group or empty vector group are depicted by * or ^#^, respectively. ***P < 0.001, ^#^*P* < 0.05, and ^###^*P* < 0.001. Con control group, Anlo anlotinib group. Data are shown as mean ± SD and representative of three independent experiments.

To measure the half-life of c-Myc in the presence of anlotinib, NCI-H929 cells were co-treated with cycloheximide (CHX) to block de novo protein synthesis. The result showed that anlotinib reduced the half-life of c-Myc protein by 2-fold (Fig. 5A). The increased turnover of c-Myc protein after anlotinib treatment could be rescued by proteasome inhibitor MG-132, suggesting that the destruction of c-Myc is mediated by the ubiquitin–proteasome system (Fig. 5B). We found that the level of ubiquitinated c-Myc species increased upon anlotinib treatment (Fig. 5C). In addition, anlotinib triggered the dephosphorylation of c-Myc at S62 and phosphorylation at T58 (Fig. S4). Whereas, anlotinib did not affect the interaction between c-Myc and its partner protein Max (Fig. S5). To clarify the molecular basis for the inhibition of c-Myc induced by anlotinib, we evaluated the possibility of c-Myc protein as a cellular direct target of anlotinib. The results of CETSA showed that the thermal stability of c-Myc protein was tapered by anlotinib in a temperature-dependent and dose-dependent manner (Figs. 5D, E and S6). To further confirm the interaction between anlotinib and c-Myc, DARTS technology was performed. As expected, anlotinib significantly protected c-Myc protein from digestion by pronase, indicating that anlotinib interacts directly with c-Myc in vitro (Fig. 5F). Moreover, we found that forced overexpression of c-Myc in NCI-H929 by lentiviral infection rescued, in part, the apoptosis induced by anlotinib (Fig. 5G). Conversely, the shRNA silencing of c-Myc increased the apoptosis rate (Fig. 5H). Whereas, neither overexpression nor knockdown of c-Myc caused significant changes in the cell cycle distributions (Fig. S7). Overall, these results indicated that anlotinib directly interacts with c-Myc and promotes its ubiquitin proteasomal degradation, which partially contributes to the apoptosis induced by anlotinib.

### Anlotinib is cytotoxic to bortezomib-resistant MM cells

**Fig. 6:**
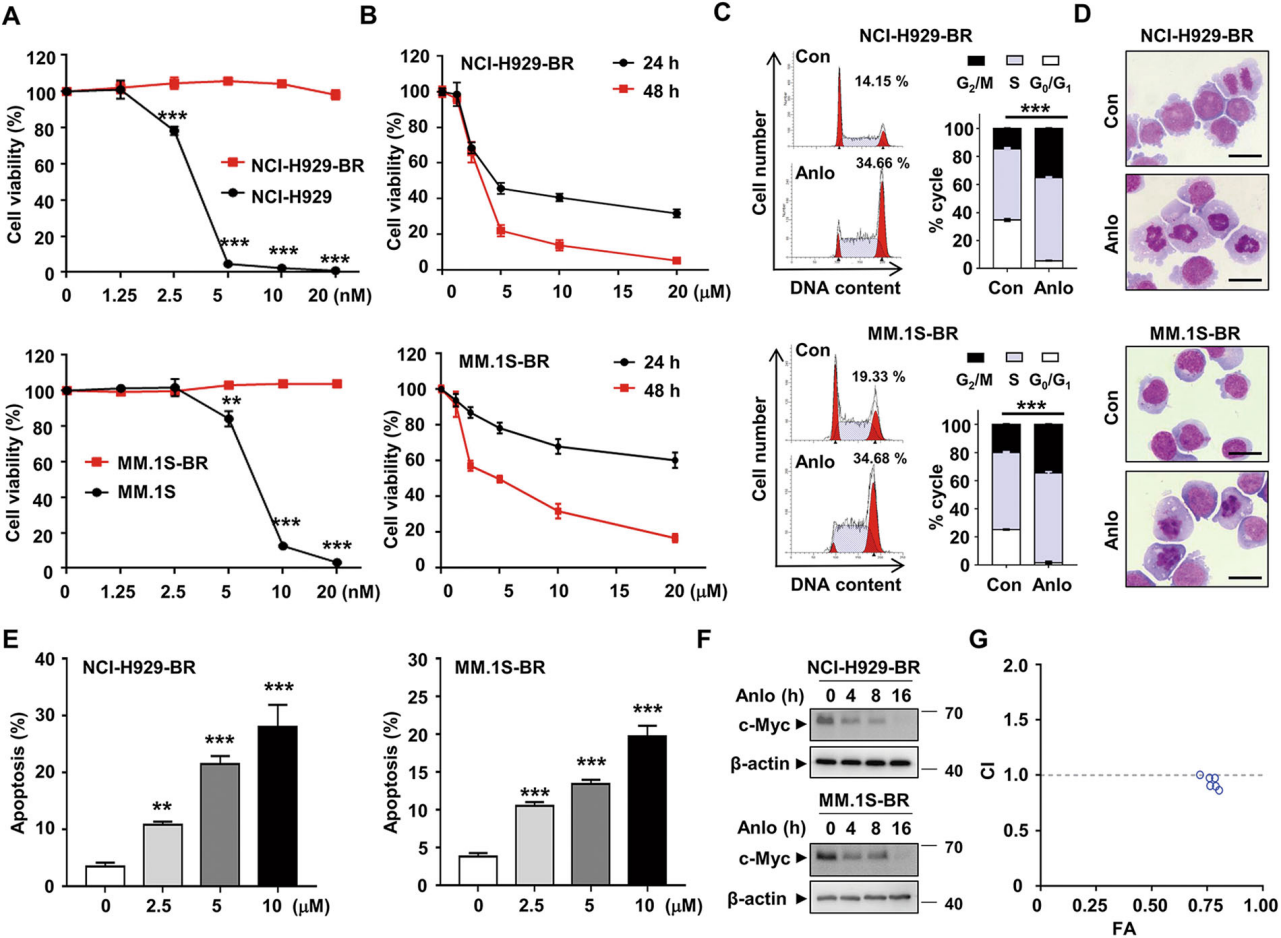
Anlotinib exhibits cytotoxicity against bortezomib-resistant MM cells. **A** NCI-H929, NCI-H929-BR, MM.1S, and MM.1S-BR cells were treated with various concentrations of bortezomib for 24 h. Cell viability was measured by CCK8 assay. **B** NCI-H929-BR and MM.1S-BR cells were treated with anlotinib (0–20 µM) for 24 and 48 h, followed by an assessment of cell viability. **C** NCI-H929-BR and MM.1S-BR cells were treated with 5 µM anlotinib for 8 h. The cell cycle was analyzed by flow cytometry. **D** The morphology of NCI-H929-BR and MM.1S-BR cells after anlotinib treatment (5 μM, 8 h) was observed using Wright staining. **E** Apoptosis of cells treated with 5 µM anlotinib for 24 h was detected by flow cytometry using Annexin V/PI staining. **F** NCI-H929-BR and MM.1S-BR cells were treated with anlotinib (5 μM) for the indicated time. Whole cell lysates were subjected to western blotting. **G** The combined effects of anlotinib and bortezomib in NCI-H929 cells were assessed using the CompuSyn software. Con: control group; Anlo: anlotinib group. ***P < 0.001. Each experiment was performed in triplicate.

To examine the cytocidal effects of anlotinib on bortezomib-resistant cells, we established NCI-H929-BR and MM.1S-BR cell lines which are effectively shown resistant to bortezomib (Fig. 6A). As showed in Fig. 6B, anlotinib remarkably suppressed the viability of NCI-H929-BR and MM.1S-BR in a dose-dependent and time-dependent manner. Similar to the bortezomib-sensitive cells, the induction of G_2_/M arrest and apoptosis was also detected in NCI-H929-BR and MM.1S-BR cells after exposure to anlotinib (Fig. 6C–E). We also analyzed the expression change of c-Myc protein and obtained similar results as shown in Fig. 6F. In addition, the CI values of anlotinib and bortezomib were nearly 1, which indicates an additive effect (Fig. 6G and Supplementary Table 1). Taken together, these data suggested that anlotinib exerts strong cytotoxicity against bortezomib-resistant MM cells.

### Antitumor efficacy of anlotinib in a MM xenograft model in vivo

**Fig. 7:**
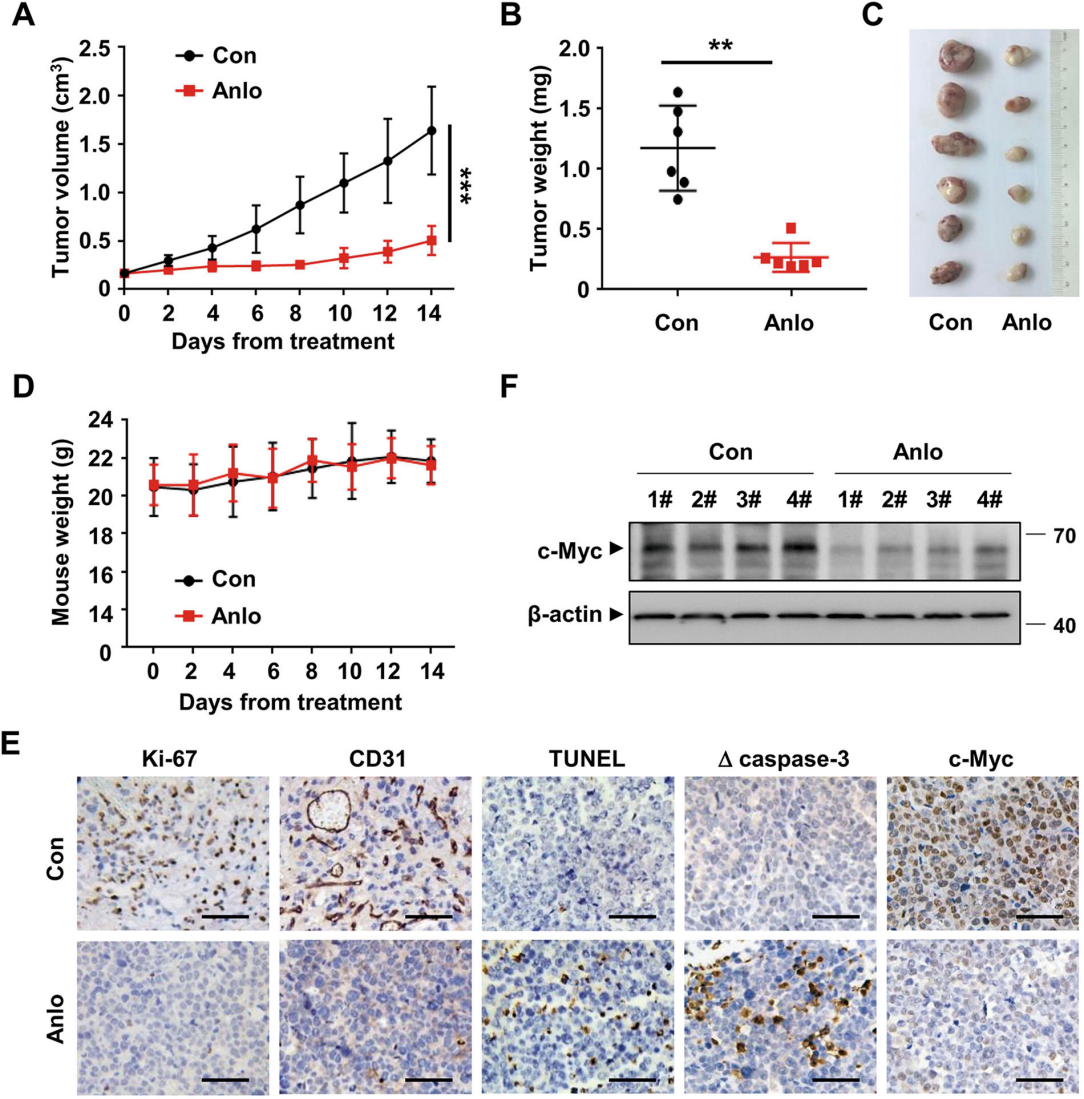
Anlotinib exhibits anti-MM activity in vivo. **A** Nude mice bearing subcutaneous NCI-H929 tumors were treated with either anlotinib (3 mg/kg) or vehicle control by intragastric administration for consecutive 14 days. Tumor size was measured every 2 days. **B** The tumor tissues were excised and weighed on day 14. **C** Gross appearance of the tumors. **D** The weight of mice was monitored every 2 days. **E** Representative IHC staining of tumor tissues. Scale bars: 50 μM. **F** Tumor tissues were lysed and subjected to western blotting to detect the protein level of c-Myc. Con control group, Anlo anlotinib group. **P < 0.01, ***P < 0.001.

Considering the encouraging anti-MM efficacy of anlotinib in vitro, we next evaluated the in vivo therapeutic potential of anlotinib in MM xenograft model. Six-week-old nude mice bearing subcutaneous NCI-H929 cells were treated with either anlotinib or vehicle control by intragastric administration for consecutive 14 days. Tumor growth and weight in the anlotinib-treated group were dramatically inhibited by approximately 69.3% and 77.2%, compared with the vehicle-treated group (Fig. 7A–C). No significant body weight loss (Fig. 7D) and treatment-related death were observed, indicating that anlotinib was well-tolerated. As shown in Fig. 7E, both the cell proliferation and vascular density were significantly suppressed, demonstrated by reduced Ki-67 staining and decreased CD31-positive microvessels, respectively. The activation of apoptosis induced by anlotinib was revealed by the increase of cleaved-caspase 3 and TUNEL-positive cells. Moreover, the IHC and western blotting analyses of the harvested tumor tissues showed that anlotinib markedly inhibited c-Myc expression in vivo (Fig. 7E, F). Taken together, these findings suggested that anlotinib has potent anti-MM activity without additional toxicity in vivo.

## Discussion

In the present study, we found that anlotinib is an exciting and promising therapeutic agent to treat MM both in vitro and in vivo. Unlike what has been described as the targets of anlotinib (e.g., VEGFR 1-3, c-Kit, PDGFR-α/β, and FGFR 1–4)^20^, we identify c-Myc for the first time as a novel direct target of anlotinib. Anlotinib displays strong cytotoxicity against bortezomib-sensitive and bortezomib-resistant MM cells, overcomes the protective effect of the bone marrow microenvironment and suppresses tumor growth in the MM mouse xenograft. Our work demonstrates the extraordinary anti-MM effect of anlotinib, which may provide a promising therapeutic strategy for human MM.

Similar to other oncogenic transcription factors, c-Myc has long been viewed as an undruggable therapeutic target due to the lack of kinase activity and intrinsically disordered structure. Several strategies have been developed to indirectly target c-Myc including blocking Myc transcription^30,31^, inhibiting bromodomain proteins^32^ and down-regulating downstream genes of MYC^33,34^. Nonetheless, the most reliable strategy is to target c-Myc directly, which abrogates downstream MYC oncogenic activities regardless of upstream alterations. Recently, small molecules that directly disrupt the c-Myc/Max interaction and DNA binding are attracting much attention^35–37^. However, many of these inhibitors are suffered from low potency and poor pharmacokinetic properties. One interesting finding in our study is the identification of c-Myc as the novel target of anlotinib, which was supported by several lines of evidence. First, the genome-wide transcriptome analysis revealed a series of downregulated target genes regulated by c-Myc, indicating c-Myc as a potential regulator in anlotinib-treated MM cells. Second, anlotinib markedly inhibited c-Myc expression in MM cells without effect on its mRNA levels. Third, the CETSA and DARTS assay confirmed the direct interaction between anlotinib and c-Myc. In 2013, Pär Nordlund et al. introduced CETSA which is a well-established assay for measurements of physical interactions between the protein and a ligand. It is based on the principle that heat could induce the unfolding and precipitation of proteins. Pär Nordlund proposed that the protein stability will increase in presence of the ligand. In our opinion, the thermal stability of proteins upon ligand binding may decrease as well, which depends on whether the binding state between ligand and protein promotes or hinders the unfolding and precipitation of the protein. The destabilization of the protein upon ligand binding has also been reported in several literatures^38,39^. DARTS is a label-free method for target identification of bioactive small molecules, which is based on the concept that the protease susceptibility of the target protein will decrease upon drug binding^25^. DARTS result demonstrated that anlotinib could directly bind to c-Myc, rendering it to be resistant to pronase treatment probably because of conformational changes induced by tight binding of anlotinib. More intriguingly, anlotinib triggered the S62 dephosphorylation and T58 phosphorylation of c-Myc, and promoted the ubiquitin proteasome-mediated degradation of c-Myc, which partially contributes to anlotinib-induced apoptosis in MM cells. Although we cannot fully clarify why c-Myc protein degradation occurs after anlotinib binding, it is possible that c-Myc protein conformation is changed upon anlotinib binding, and thus increasing its association with E3 ubiquitin ligases or decreasing its association with deubiquitinases, which subsequently enhances the proteasome-dependent degradation of c-Myc. In addition to MM, c-Myc is overexpressed and activated in many other cancers. Interestingly, we have investigated the effects of anlotinib on other cancer cell lines (HL60, SU-DHL-2, and OCI-Ly3) with strong c-Myc dependency. The results showed decreased cell viability of these cells with down-regulation of c-Myc expression after anlotinib treatment, which are similar to the effect of anlotinib in MM cells (Fig. S8). Recent preclinical and ongoing clinical trials have demonstrated promising anti-tumor activities of anlotinib against diverse malignant tumors^21–23^. More studies are needed to investigate whether the degradation of c-Myc contributes to the effects of anlotinib or the expression of c-Myc affects the sensitivity of anlotinib.

Additionally, we found that overexpression of c-Myc just partially rescued the apoptosis induced by anlotinib in MM, indicating there are other mechanisms. Although MM lacks disease-defining tyrosine kinase fusion genes, it is characterized by the activation of multiple tyrosine kinase signaling cascades. The tumor-specific driver aberrations of MM, such as genetic abnormalities and microenvironment-driven deregulations, promote disease progression partly mediated by signaling molecules. The signaling molecules include IL-6^40^, transforming growth factor-beta (TGF-β)^41^, VEGF^42^, insulin-like growth factor 1 (IGF1)^40^, hepatocyte growth factor (HGF)^43^, tumor necrosis factor-alpha (TNF-α)^44^, Fms-like tyrosine kinase 3 (FLT3)^45^, and so on. These cytokines function upstream of RTKs and regulate multiple downstream signaling cascades including the Ras/Raf/MEK/MAPK, PI3K/Akt/mTOR, JAK/STAT, and NF-kB pathway. Thus, targeting the activated RTKs signaling cascades with TKIs may be a promising therapeutic strategy against MM. Currently, numerous TKIs are being studied in MM, especially in the relapsed/refractory setting^46^. Nevertheless, so far, no TKIs have been approved for MM treatment, indicating the need for preclinical research to identify novel compounds. The well-recognized molecular targets for anlotinib are VEGFR1–3, c-Kit, PDGFR-α/β, and FGFR1–4, the majority of which are abnormally active in MM^47–49^. Considering the core downstream pathways that RTKs signaling cascades converge at, we investigated the effects of anlotinib on those pathways and found that anlotinib inhibits the MAPK, PI3K/Akt/mTOR, JAK/STAT, and NF-κB pathways in MM cells. Moreover, accumulating evidence indicates that bone marrow microenvironment mediates drug resistance by offering protection against cytotoxic agents, leading to the selection and outgrowth of malignant cells under the selective pressure of drug exposure^50,51^. Our results show that anlotinib antagonizes the protective effect of the bone marrow microenvironment and effectively induced cytotoxicity against bortezomib-resistant MM cells.

In summary, anlotinib exerts promising anti-MM activity in cultured and primary MM cells, as well as in MM mouse xenograft model. Directly targeted degradation of c-Myc is a novel mechanism by which anlotinib causes apoptosis in MM cells. Moreover, anlotinib could effectively induce cytotoxicity against bortezomib-sensitive and bortezomib-resistant MM cells, and antagonize the protective effect of the bone marrow microenvironment. These results provide a basis for future clinical trials to investigate the efficacy and safety of anlotinib in the treatment of MM.

## Supplementary information

Fig. S1

Fig. S2

Fig. S3

Fig. S4

Fig. S5

Fig. S6

Fig. S7

Fig. S8

supplementary figure legend

supplementary table 1

## Data Availability

The data that support the findings of this study are available from the corresponding author upon reasonable request.
