# Supplementary figures and images for: Directly targeting c-Myc contributes to the anti-multiple myeloma effect of anlotinib
Source: Cell Death Dis. 2021 Apr 14;12(4):396. doi: 10.1038/s41419-021-03685-w (PMC8046985; doi:10.1038/s41419-021-03685-w)

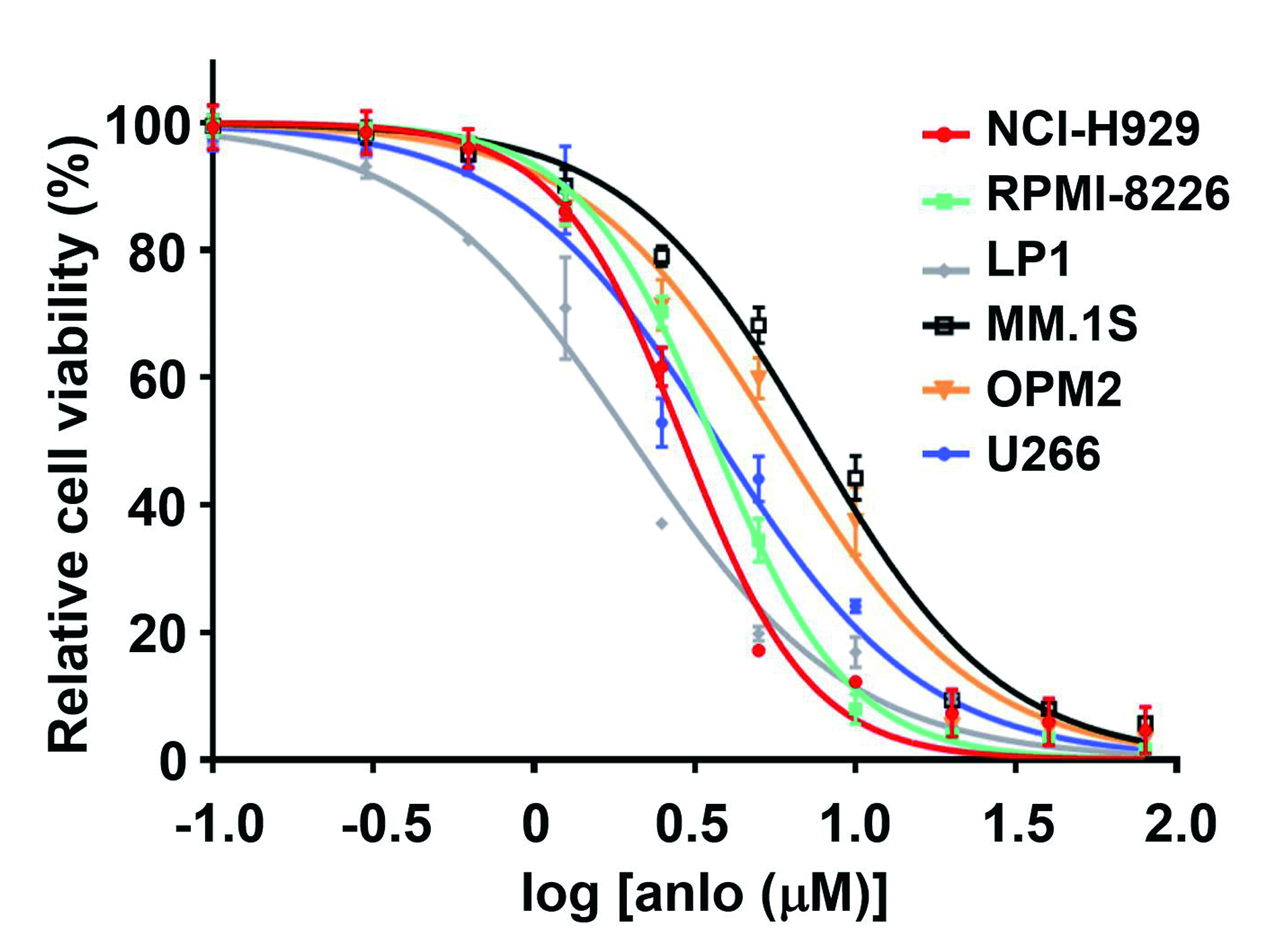

Supplement: Supplementary file 1 — Fig. S1 [file 41419_2021_3685_MOESM1_ESM.tif]

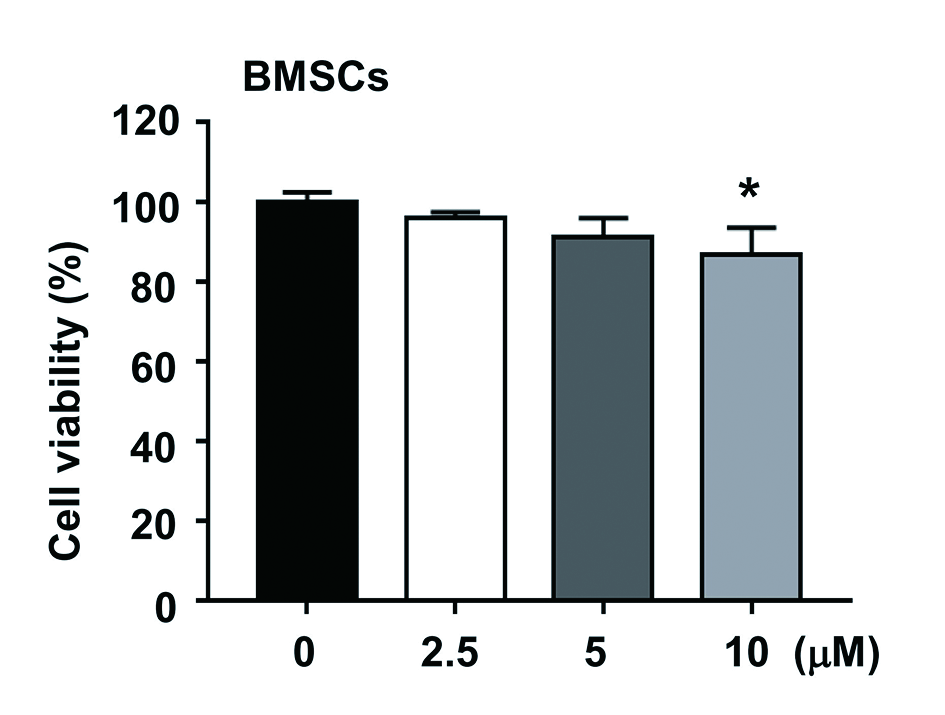

Supplement: Supplementary file 2 — Fig. S2 [file 41419_2021_3685_MOESM2_ESM.tif]

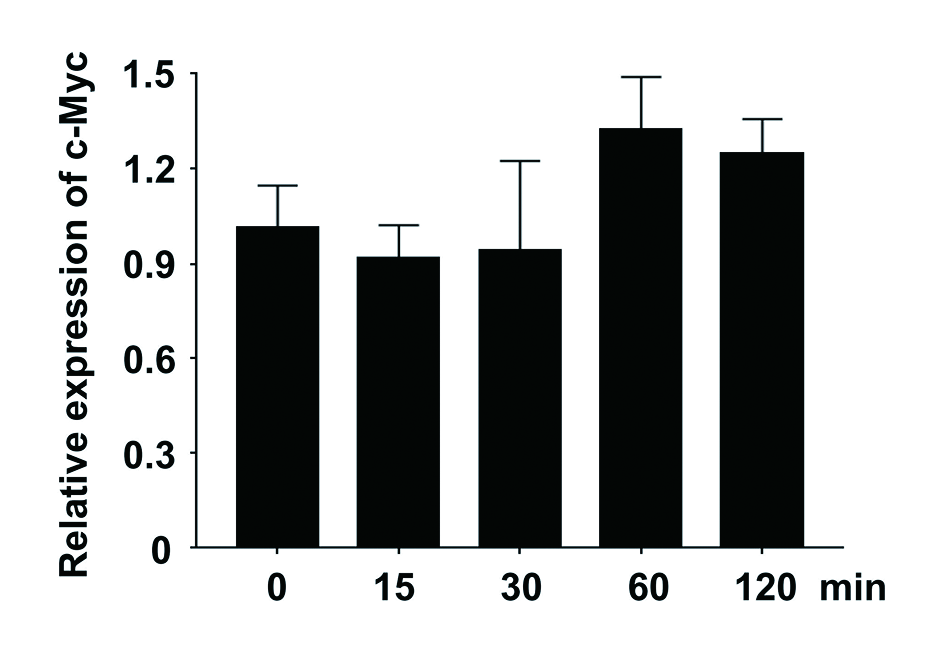

Supplement: Supplementary file 3 — Fig. S3 [file 41419_2021_3685_MOESM3_ESM.tif]

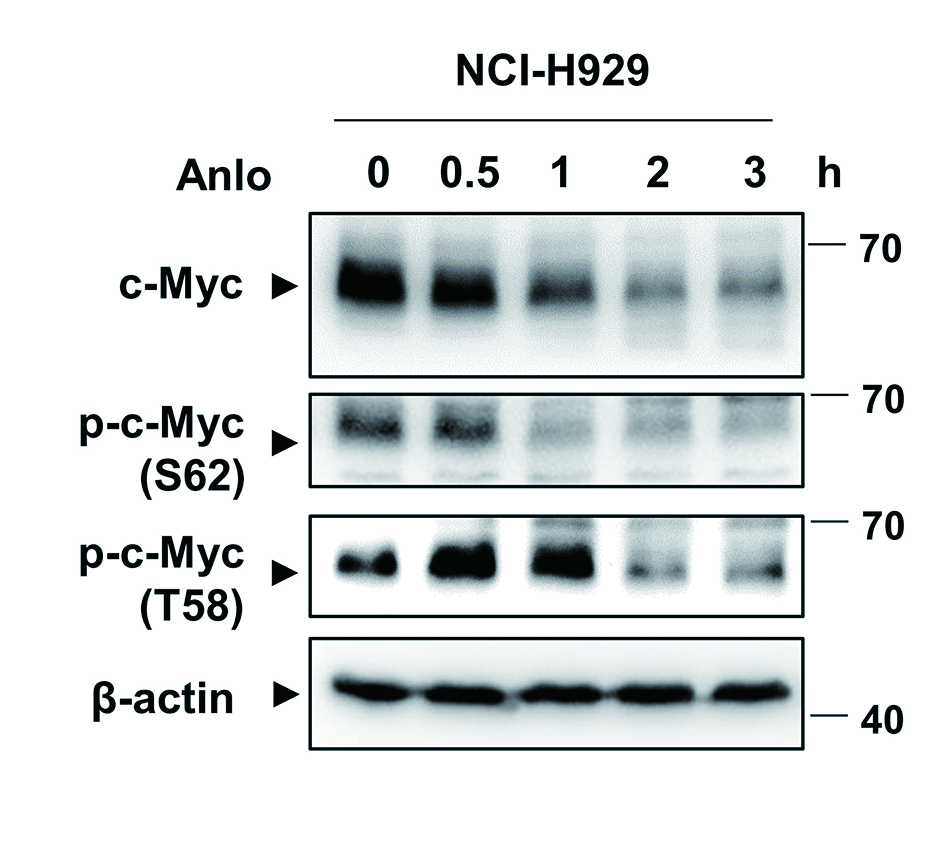

Supplement: Supplementary file 4 — Fig. S4 [file 41419_2021_3685_MOESM4_ESM.tif]

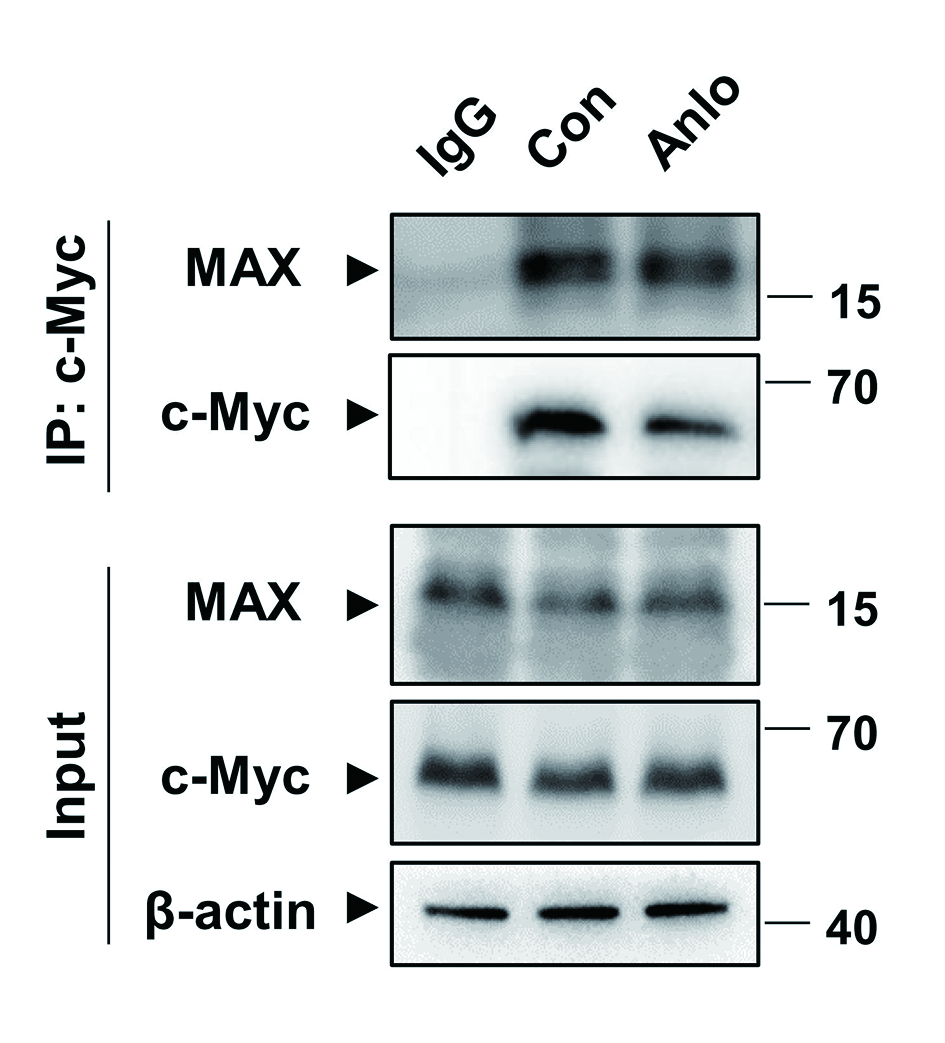

Supplement: Supplementary file 5 — Fig. S5 [file 41419_2021_3685_MOESM5_ESM.tif]

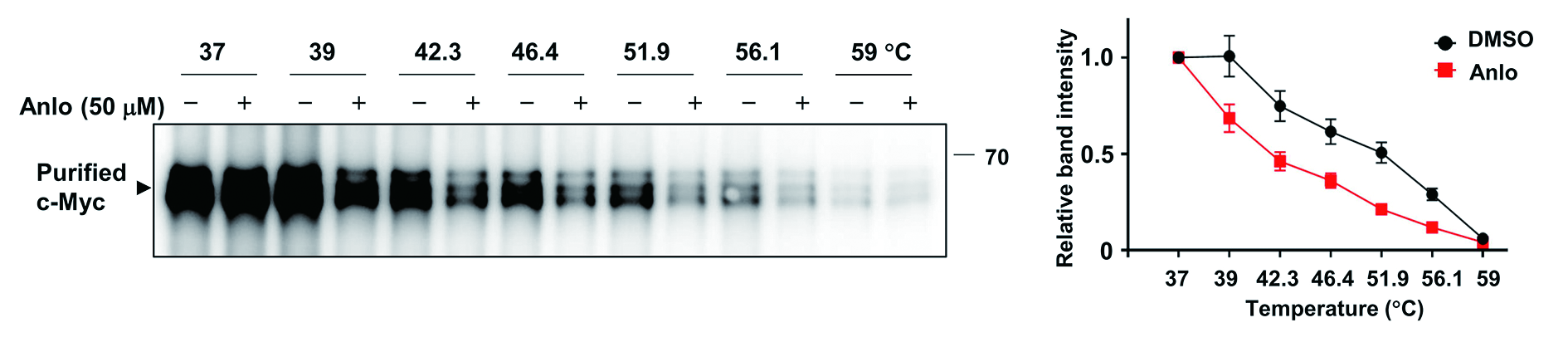

Supplement: Supplementary file 6 — Fig. S6 [file 41419_2021_3685_MOESM6_ESM.tif]

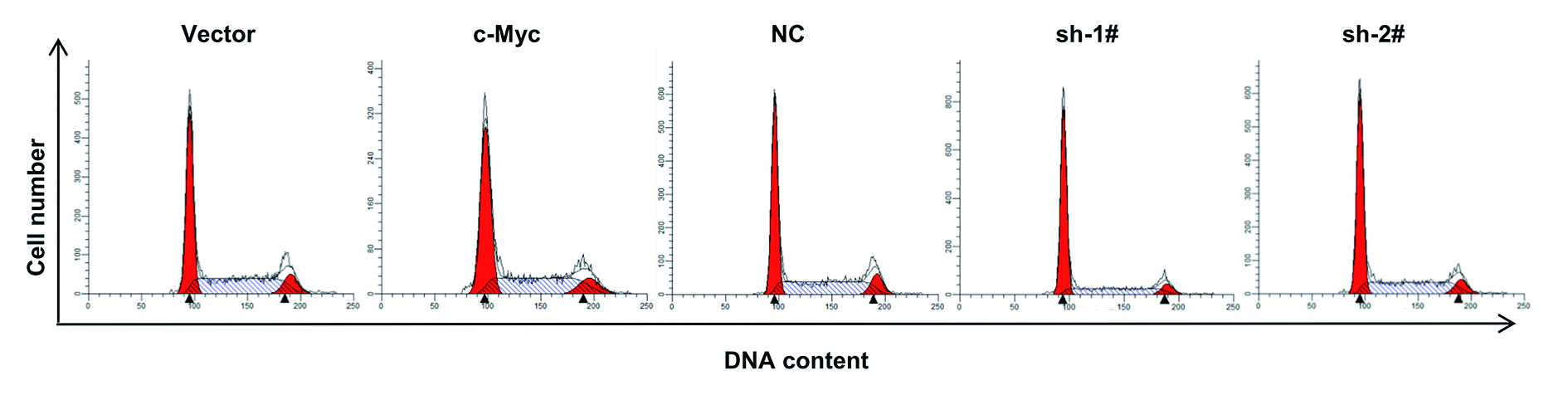

Supplement: Supplementary file 7 — Fig. S7 [file 41419_2021_3685_MOESM7_ESM.tif]

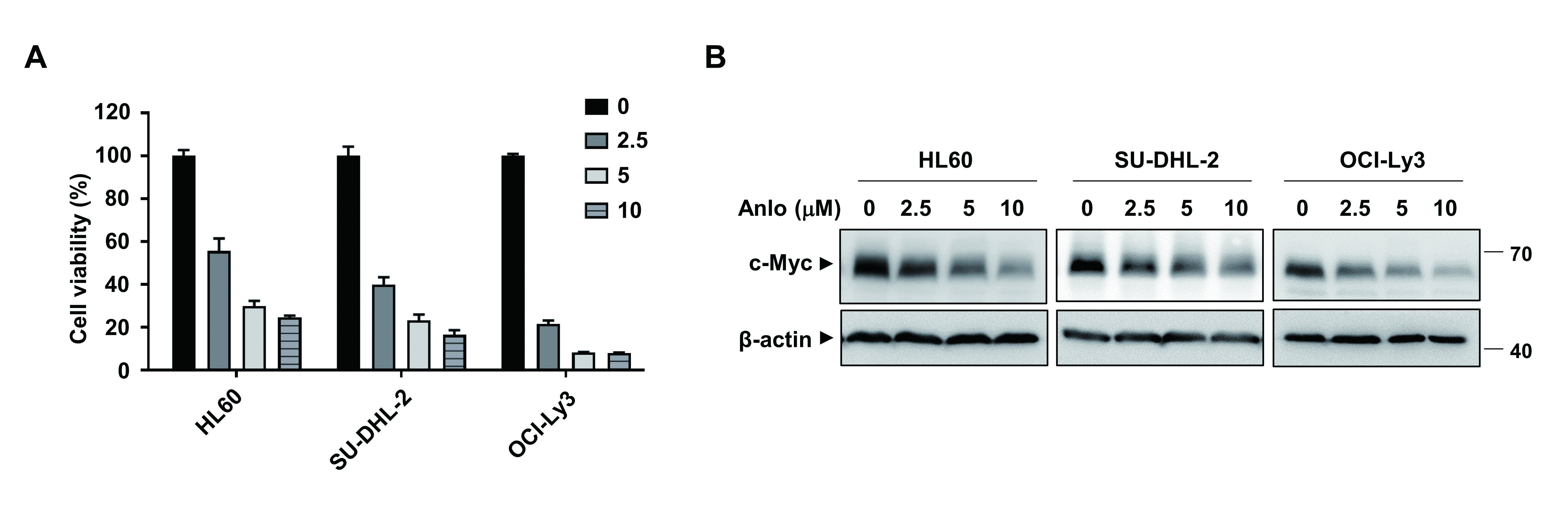

Supplement: Supplementary file 8 — Fig. S8 [file 41419_2021_3685_MOESM8_ESM.tif]
