# Supplementary material for: Directly targeting c-Myc contributes to the anti-multiple myeloma effect of anlotinib
Source: Cell Death Dis. 2021 Apr 14;12(4):396. doi: 10.1038/s41419-021-03685-w (PMC8046985; doi:10.1038/s41419-021-03685-w)
Supplement: Supplementary file 9 — supplementary figure legend [file 41419_2021_3685_MOESM9_ESM.docx]

**Fig. S1.** The graph generated using GraphPad Prism software for IC50 calculations of anlotinib based on CCK8. The MM cell lines (NCI-H929, RPMI-8226, LP1, MM.1S, OPM2 and U266) were treated with different concentrations of anlotinib for 48 h and analyze by CCK8 test. The data generated were plotted with GraphPad Prism software. Each experiment was performed in triplicate.

**Fig. S2.** Analysis of the cell viability of BMSCs after treated with anlotinib (0-10 μM) for 48 h.

**Fig. S3.** The mRNA levels of c-Myc in NCI-H929 cells after treated with anlotinib for indicated times were analyzed by qRT-PCR.

**Fig. S4.** NCI-H929 cells were treated with 5 μM anlotinib for the indicated time. The cell lysates were subjected to western blotting using c-Myc, p-c-Myc (S62), p-c-Myc (T58), and β-actin antibody. Anlo: anlotinib.

**Fig. S5.** NCI-H929 cells were treated with 5 μM anlotinib for 0.5 h. The cell lysates were immunoprecipitated with anti-c-Myc antibody and then probed with anti-MAX antibody. Con: control group; Anlo: anlotinib group.

**Fig. S6.** The binding between anlotinib (50 μM) and purified c-Myc protein was examined by the CETSA method at different temperatures. The indicated proteins were evaluated by western blotting (left). CETSA curves of c-Myc were determined in the absence and presence of anlotinib. Each band intensity of c-Myc in DMSO or anlotinib group was normalized with respect to that obtained at the lowest temperature. Anlo: anlotinib.

**Fig. S7.** The effects of c-Myc knockdown or overexpression on the cell cycle were evaluated by flow cytometry.

**Fig. S8.** The HL60, SU-DHL-2 and OCI-Ly3 cells were treated with anlotinib (0-10 μM). The cell viability was detected by CCK8 and the cell lysates were immunoprecipitated with anti-c-Myc antibody.
