# Supplementary material for: Directly targeting c-Myc contributes to the anti-multiple myeloma effect of anlotinib
Source: Cell Death Dis. 2021 Apr 14;12(4):396. doi: 10.1038/s41419-021-03685-w (PMC8046985; doi:10.1038/s41419-021-03685-w)
Supplement: Supplementary file 10 — supplementary table 1 [file 41419_2021_3685_MOESM10_ESM.doc]

**Table S1.** **The CI values of anlotinib and bortezomib in NCI-H929 cells**

| Anlo (μM) | BZT (nM) | CI |
| --- | --- | --- |
| 1.5 | 2.75 | 0.86264 |
| 2.0 | 2.75 | 0.90421 |
| 3.0 | 2.75 | 0.89993 |
| 1.5 | 3.0 | 0.97499 |
| 2.0 | 3.0 | 1.02403 |
| 2.5 | 3.0 | 0.97255 |
